# An *Alphavirus* E2 Membrane-Proximal Domain Promotes Envelope Protein Lateral Interactions and Virus Budding

**DOI:** 10.1128/mBio.01564-17

**Published:** 2017-11-07

**Authors:** Emily A. Byrd, Margaret Kielian

**Affiliations:** Department of Cell Biology, Albert Einstein College of Medicine, Bronx, New York, USA; Johns Hopkins Bloomberg School of Public Health

**Keywords:** virus assembly, virus budding, alphavirus, virus entry, virus fusion, virus structure

## Abstract

Alphaviruses are members of a group of small enveloped RNA viruses that includes important human pathogens such as Chikungunya virus and the equine encephalitis viruses. The virus membrane is covered by a lattice composed of 80 spikes, each a trimer of heterodimers of the E2 and E1 transmembrane proteins. During virus endocytic entry, the E1 glycoprotein mediates the low-pH-dependent fusion of the virus membrane with the endosome membrane, thus initiating virus infection. While much is known about E1 structural rearrangements during membrane fusion, it is unclear how the E1/E2 dimer dissociates, a step required for the fusion reaction. A recent *Alphavirus* cryo-electron microscopy reconstruction revealed a previously unidentified D subdomain in the E2 ectodomain, close to the virus membrane. A loop within this region, here referred to as the D-loop, contains two highly conserved histidines, H348 and H352, which were hypothesized to play a role in dimer dissociation. We generated Semliki Forest virus mutants containing the single and double alanine substitutions H348A, H352A, and H348/352A. The three D-loop mutations caused a reduction in virus growth ranging from 1.6 to 2 log but did not significantly affect structural protein biosynthesis or transport, dimer stability, virus fusion, or specific infectivity. Instead, growth reduction was due to inhibition of a late stage of virus assembly at the plasma membrane. The virus particles that are produced show reduced thermostability compared to the wild type. We propose the E2 D-loop as a key region in establishing the E1-E2 contacts that drive glycoprotein lattice formation and promote *Alphavirus* budding from the plasma membrane.

## IMPORTANCE

*Alphavirus* infection causes severe and debilitating human diseases for which there are no effective antiviral therapies or vaccines. In order to develop targeted therapeutics, detailed molecular understanding of the viral entry and exit mechanisms is required. In this report, we define the role of the E2 protein juxtamembrane D-loop, which contains highly conserved histidine residues at positions 348 and 352. These histidines do not play an important role in virus fusion and infection. However, mutation of the D-loop histidines causes significant decreases in the assembly and thermostability of *Alphavirus* particles. Our results suggest that the E2 D-loop interacts with the E1 protein to promote *Alphavirus* budding.

## INTRODUCTION

Alphaviruses are members of a group of enveloped positive-sense RNA viruses that includes clinically important viruses such as Chikungunya virus (CHIKV), Mayaro virus, Ross River virus, and the Western equine encephalitis viruses, Eastern equine encephalitis viruses, and Venezuelan equine encephalitis viruses (VEEV) (reviewed in references 1, 2, and 3). Most commonly transmitted by mosquitoes, *Alphavirus* infection causes a significant global disease burden, with CHIKV alone infecting 7.5 million people in only a 5-year time period, resulting in several thousand deaths (4–7). *Alphavirus* infection can cause a wide array of symptoms, including fever and encephalitis or fever with debilitating arthralgia, that can last for weeks to years (2, 4). There are currently no vaccines or effective therapies to combat these diseases.

Alphaviruses are spherical particles with very organized structures and high specific infectivity (reviewed in references 1, 8, and 9). Both the capsid and envelope proteins are arranged with T=4 icosahedral symmetry. The internal nucleocapsid core contains a single copy of the RNA genome surrounded by a lattice of 240 copies of the capsid protein. This core is enveloped by the virus lipid bilayer, which contains a lattice composed of 240 copies of the transmembrane E1 and E2 proteins, closely associated as heterodimers and further organized into 80 trimeric spikes. E2 covers much of the underlying E1 protein and is the major target of neutralizing antibodies (Abs) and is responsible for virus-receptor interactions.

*Alphavirus* infection occurs via receptor binding at the plasma membrane, internalization by clathrin-mediated endocytosis, and low-pH-triggered fusion of the virus membrane with the endosome membrane (1, 10). Fusion is driven by viral membrane protein E1, a class II membrane fusion protein (11, 12). E1 undergoes conformational changes within the acidic environment of the early endosome, resulting in insertion of the E1 fusion loop into the target membrane, E1 trimerization, refolding to a hairpin conformation, and formation of a fusion pore. The nucleocapsid is thereby released into the cytoplasm, where it dissociates to release the viral RNA for subsequent translation and replication.

The viral structural proteins are generated from a polyprotein, with E2 initially synthesized as a precursor termed p62 that dimerizes with E1 in the endoplasmic reticulum (1). p62 is processed by cellular furin late in the secretory pathway, generating mature E2 and peripheral E3. Virus budding occurs at the plasma membrane in a process that requires the 1:1 interaction of the short E2 cytoplasmic domain with a hydrophobic pocket on the capsid protein (13–15).

The structures of the p62/E2-E1 dimer (9, 16) reveal the extensive interactions of these proteins, while functional studies demonstrate the importance of these interactions at critical steps during virus entry and exit (reviewed in reference 17). During virus biogenesis, the stable p62-E1 dimer promotes E1 folding and transport to the plasma membrane (1, 18). Following p62 processing by furin, E3 remains bound in the low-pH environment of the late secretory pathway, thus stabilizing the dimer and protecting E1 from acid inactivation during transport (19, 20). The release of E3 in the neutral pH extracellular environment then primes the virus for subsequent low-pH-triggered fusion in the endosome (19). A critical step in the virus fusion reaction is the dissociation of the E1-E2 heterodimer (18, 21). Dimer dissociation is triggered by low pH in a stepwise process, with an initial rearrangement of E2 domain B uncapping the E1 fusion loop (9, 16, 22). To complete the fusion reaction, E1 proteins must interact laterally and refold to the trimeric hairpin, suggesting that complete dissociation of E2 from E1 and disassembly of the trimeric spike complexes are required. The mechanism of this dimer dissociation is currently unknown.

**FIG 1:**
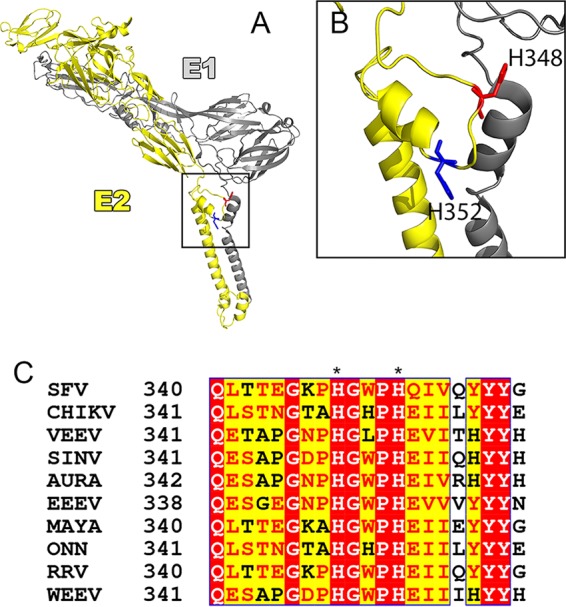
The E2 D-loop and the highly conserved H348 and H352 histidines. (A) Model of the VEEV surface glycoproteins showing E1 in gray and E2 in yellow, with H348 shown in red and H352 in blue (residues are numbered as for SFV [VEEV, H349 and H53]) (PDB accession no. 3J0C). (B) Expanded view of the boxed area in panel A showing the E2 D-loop with H348 and H352. The images in panels A and B were prepared using PyMOL software (the PyMOL Molecular Graphics System, v1.2r2; Schrödinger, LLC). (C) Alignment of E2 proteins from members of the *Alphavirus* genus, demonstrating that H348 and H352 are highly conserved (numbering as described for SFV). Red highlighting indicates high sequence conservation, and yellow highlighting indicates some sequence conservation, with conserved residues shown in red text. Alignment was performed using NPS@ (58) with CLUSTAL W (59). Alignment formatting was carried out using ESPript (60). SINV, Sindbis virus; AURA, Aura virus; EEEV, Eastern equine encephalitis virus; MAYA, Mayaro virus; ONN, O'nyong-nyong virus; RRV, Ross River virus; WEEV, Western equine encephalitis virus.

A recent cryo-electron (cryo-EM) microscopy reconstruction of Venezuelan equine encephalitis virus (VEEV) revealed a previously unidentified D subdomain in the ectodomain of E2 (VEEV E2 residues 342 to 367) (23). This juxtamembrane region contained a loop (here referred to as the D-loop) and a helix. The D-loop lies close to the virus membrane and contains two highly conserved histidines, H348 and H352 (residue numbering as in Semliki Forest virus [SFV]; see Fig. 1). It was proposed that the low pH of the endosome during virus entry would result in protonation of H348 and H352, allowing interaction of these residues with the negatively charged lipid head groups of the virus membrane. This interaction could potentially anchor E2 or cause a conformational rearrangement, promoting the dissociation of E1 and E2.

Here we addressed the role of E2 D-loop residues H348 and H352 using the *Alphavirus* SFV as a model system. Our data do not support the idea of a key role of the D-loop in dimer dissociation during virus fusion. Instead, we identified an unexpected budding defect in H348/352A mutants. This defect is due to the disruption of conserved contacts with the E1 glycoprotein that are necessary for particle budding. We propose that H348 and H352 are critical for the formation of the E1/E2 lattice at the plasma membrane, thus highlighting the importance of glycoprotein interactions in virus budding.

## RESULTS

### Initial characterization of E2 H348 and H352 mutants.

In order to test the role of H348 and H352 in the E2 glycoprotein D-loop, we created single and double alanine substitutions in the pSP6-SFV4 infectious clone (24), subjected the viral RNAs to *in vitro* transcription, and electroporated them into BHK-21 cells to test the phenotypes. These mutants are referred to here as H348A, H352A, and H348/352A and the wild-type virus produced from the SFV infectious clone as WT. Immunofluorescence of cells fixed at 8 h postelectroporation (hpe) showed efficient cell surface expression of E1 and E2 for all mutants (see Fig. S1 in the supplemental material). Analysis of cocultures of electroporated cells and nonelectroporated cells showed that by 24 hpe all of the mutants were able to mediate secondary infection (data not shown).

10.1128/mBio.01564-17.1 FIG S1  Cell surface expression of E1 and E2. BHK cells were electroporated with WT or mutant RNAs, fixed 8 h postelectroporation, and stained for cell surface expression of E1 (A) or E2 (B) using specific MAbs. The nuclei were stained with Hoechst. Scale bars represent 10 μm. Images are representative of results of 2 independent experiments. Download FIG S1, JPG file, 0.8 MB. Copyright © 2017 Byrd and Kielian. 2017 Byrd and Kielian This content is distributed under the terms of the Creative Commons Attribution 4.0 International license.

**FIG 2:**
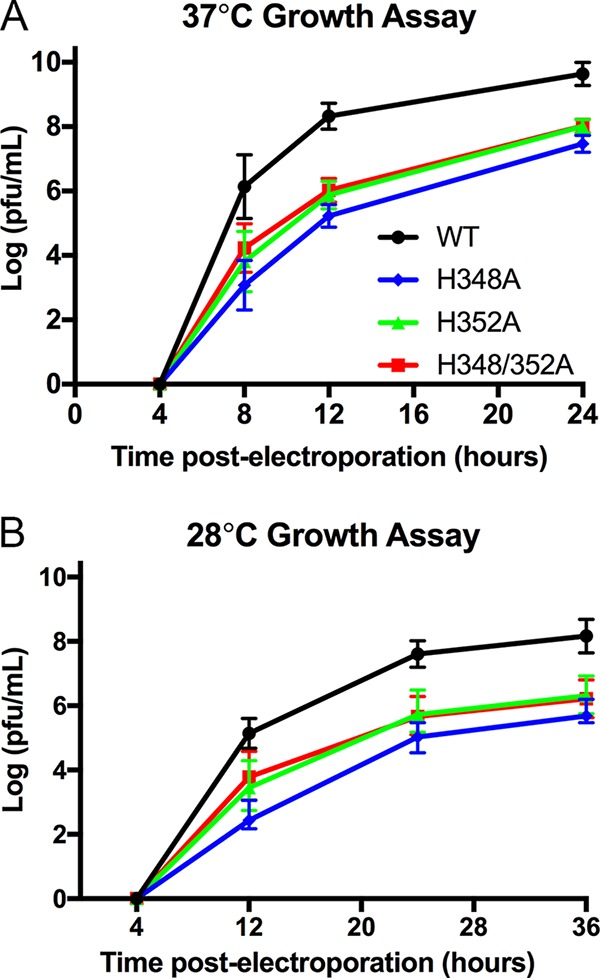
Growth properties of WT and mutant viruses. BHK cells were electroporated with WT or mutant viral RNA and incubated at 37°C (A) or 28°C (B). Cell media were collected at the indicated times, and virus production was quantitated by plaque assay. (A) Growth kinetics at 37°C. The graph shows averages and standard deviations of data from 3 to 4 independent experiments; two independent clones of each mutant were assayed in each experiment. At the time points of 8, 12, and 24 h, results from all 3 mutants were significantly different from WT results (*P* < 0.0001). The titers produced by the H348A, H352A, and H348/352A mutants were not significantly different at 24 h (*P* > 0.2). (B) Growth kinetics at 28°C. The graph shows averages of data from 2 independent experiments, each performed using two independent clones for each mutant. The bars indicate the ranges.

We then performed growth assays by electroporation of WT and mutant RNAs into BHK cells. Growth of all three mutants at 37°C was significantly inhibited, with a 1.6-to-2-log reduction in growth compared to the WT at 24 h (Fig. 2A). The decreases in growth of the three mutants were similar, with no significant differences among their titers at 24 h and thus no apparent additive effects of the mutations (Fig. 2A). While some *Alphavirus* mutants with impaired virus production are rescued by growth at 28°C (25, 26), the growth of the H348A, H352A, and H348/352A mutants remained inhibited when RNA-electroporated BHK cells were incubated at 28°C (Fig. 2B).

### Role of H348 and H352 in virus entry.

**FIG 3:**
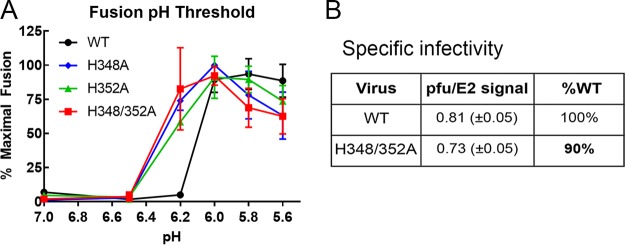
Effects of H348 and H352 mutations on virus fusion and infectivity. (A) WT and mutant viruses were prebound to BHK cells for 90 min on ice and then treated with media at the indicated pH for 3 min at 37°C to trigger virus fusion with the plasma membrane. Cells were cultured at 28°C for 16 h in the presence of 20 mM NH_4_Cl to prevent secondary infection. The percentage of infected cells was quantitated by immunofluorescence and normalized to the maximal fusion for each virus. The graph shows averages and standard deviations of data from 3 independent experiments. The difference between the results from the WT strain and all three mutants at pH 6.2 is statistically significant (*P* < 0.0001). (B) The specific infectivity of WT and mutant viruses was calculated by determining the ratio of infectious virus (as quantitated by plaque assay) to the number of virus particles (as quantitated by Western blotting of the E2 glycoprotein). The difference in specific infectivity between WT and H348/352A was not statistically significant (*P* > 0.1). Data shown represent averages and standard deviations of data from 3 independent experiments.

To test the hypothesis that H348 and H352 play an important role in E2 dissociation from E1 during virus entry, we first determined the pH threshold of fusion of the mutant viruses, since changes in dimer dissociation shift the pH requirements for fusion (18, 21, 27, 28). Mutant and WT viruses were bound to BHK cells and pulsed at low pH to trigger virus-plasma membrane fusion, and the resultant infection was quantitated by immunofluorescence. Fusion of WT SFV had a pH threshold of ~6.0 in this assay, while the mutant viruses all had a pH threshold of ~6.2 (Fig. 3A). Although this difference was experimentally significant, such minor shifts in fusion pH threshold have not been observed to affect virus growth or infectivity (see, e.g., references 29 and 30, and we note that the difference is based on a single pH point. A small increase in the mutant pH threshold could reflect somewhat reduced stability in the E1/E2 dimer of the mutant virus particles, contrary to what would be predicted by the dimer dissociation hypothesis.

Given that the three mutants have similar properties, our further investigations focused on the H348/352A double mutant. We determined virus-specific infectivity, since it would be decreased if virus entry and fusion were less efficient in the mutant. WT and mutant SFV stocks were harvested from 8-h infections of BHK cells, and virus-specific infectivity was calculated as the ratio of the plaque-forming unit number to the virus particle E2 protein number. No significant difference was observed between the specific infectivity of WT SFV and that of the H348/352A mutant (Fig. 3B). Taken together, our results indicate that although mutation of the D-loop histidine residues strongly inhibited virus growth, it did not significantly affect dimer dissociation or virus infectivity.

### Induction of intercellular extensions.

**FIG 4:**
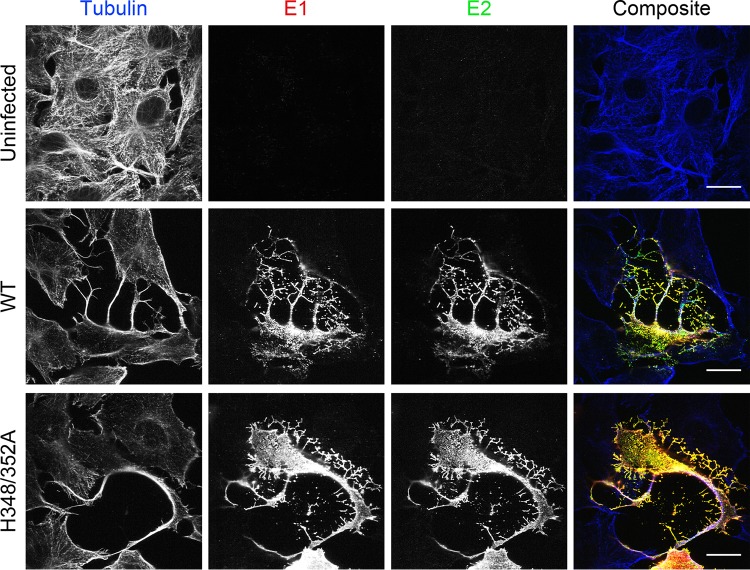
Induction of intercellular extensions by WT and mutant viruses. Vero cells were infected with WT SFV or the H348/352A mutant and fixed at 8 hpi. Cells were permeabilized; stained with antibodies to tubulin, E2, and E1; and imaged by confocal microscopy. Both the WT- and H348/352A-infected cells produced long (>10-μm), tubulin-positive intercellular extensions. Images shown are representative of 2 independent experiments. Scale bars = 20 μm.

Previous work from our laboratory and others showed that *Alphavirus* infection induces long (>10-µm) actin- and tubulin-positive intercellular extensions that can transmit virus from infected cells to uninfected cells (31–34). Induction of extensions is abrogated by mutation of a critical tyrosine residue in the E2 cytoplasmic tail, which blocks E2-capsid interaction and virus budding (31, 32). We hypothesized that structural changes in the H348/352A mutant could perturb E2-capsid interactions, thus reducing formation of intercellular extensions and cell-to-cell virus transmission. We therefore evaluated the formation of extensions in Vero cells infected with WT or H348/352A virus. Vero cells were optimal for these experiments as their flat morphology permits efficient imaging of extensions (32). Cells were fixed at 8 h postinfection (hpi); stained with antibodies against tubulin, E1, and E2; and imaged by confocal microscopy (Fig. 4). Both WT and H348/352A virus induced long, tubulin-positive extensions, arguing that the route of cell-to-cell virus transmission *per se* would not be impaired by the D-loop mutations. Similar results were obtained using WT- and mutant-infected BHK cells (data not shown).

### Assembly of H348/352A virus particles.

**FIG 5:**
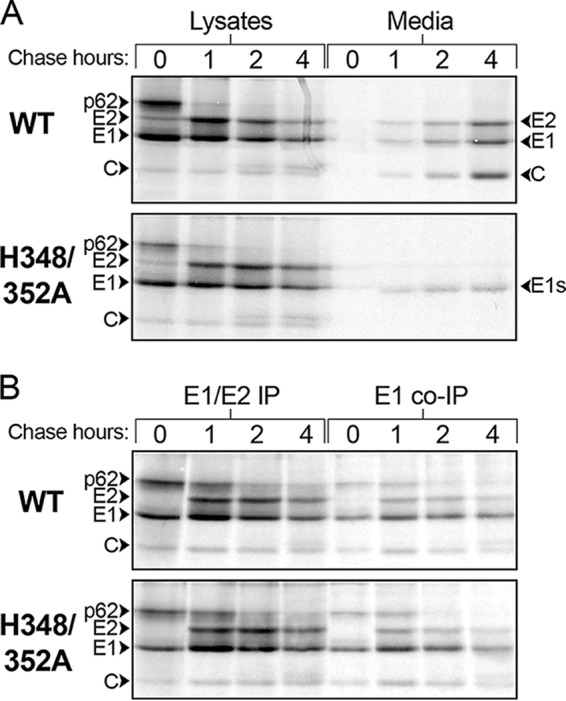
Assembly and dimer stability of WT and mutant viruses. (A) Virus assembly. BHK cells were infected with WT SFV or the H348/352A mutant for 5 h, pulse-labeled for 30 min with [^35^S]methionine/cysteine, and chased for the indicated times. At each time point, the cells were lysed and immunoprecipitated with a pAb (to E1 and E2), and the virus in the chase medium was retrieved by IP in the absence of detergent. Samples were analyzed by SDS-PAGE and fluorography. The positions of the viral structural proteins and E1s, the soluble truncated form of E1, are indicated. (B) Dimer stability. BHK cells were infected, pulse-labeled, and chased as described for panel A. The cell lysates were immunoprecipitated with a pAb to E1 and E2 or a MAb to E1. The results shown in panels A and B are representative of 2 independent experiments.

The lack of effects of the D-loop mutations on particle infectivity and extension formation suggested that the mutant growth defects could be due to inhibition of particle assembly. To test this, BHK cells were infected, pulse-labeled with [^35^S]methionine/cysteine at 5 hpi, and chased in the absence of label for 0 to 4 h. The cell media and lysates were collected, immunoprecipitated with a polyclonal Ab (pAb) against E1 and E2, and evaluated by SDS-PAGE. The cell lysate samples showed that WT- and mutant-infected cells produced the viral structural proteins and processed p62 to E2 with similar efficiencies and kinetics (Fig. 5A). The cell medium samples were immunoprecipitated in the absence of detergent to allow retrieval of intact virus particles. Increasing amounts of the viral structural proteins were recovered from the chase media of WT-infected cells with increasing chase times (Fig. 5A). In contrast, the mutant-infected cells produced negligible amounts of virus particles and primarily released E1s, a soluble fragment of E1 produced under budding-defective conditions (35, 36). In agreement with the results of growth assays at 28°C (Fig. 2B), incubation at 28°C did not rescue mutant virus assembly (data not shown). Thus, the H348/352A mutant is strongly impaired for virus assembly and this is the primary cause of its significant reduction in growth.

The p62/E2 protein acts as a chaperone for E1 folding and protects it from premature fusion in the secretory pathway (19, 20). *Alphavirus* assembly defects can occur due to failure of the E1 and E2 dimers to associate after synthesis (22) or to decreased stability of the dimers during virus budding at the cell surface (25). To test the stability of the H348/352A dimer during virus biogenesis, infected BHK cells were pulse-labeled and chased. Aliquots of the cell lysates were then immunoprecipitated with pAb to E1/E2 or with an E1 monoclonal antibody (MAb) to evaluate dimer stability by coimmunoprecipitation (Fig. 5B). The MAb to E1 retrieved comparable levels of associated p62/E2 from WT-infected and mutant-infected cells. These results suggest that the stability of the mutant E1/E2 dimer is not responsible for its assembly defect.

### Mutant budding and nucleocapsid production.

**FIG 6:**
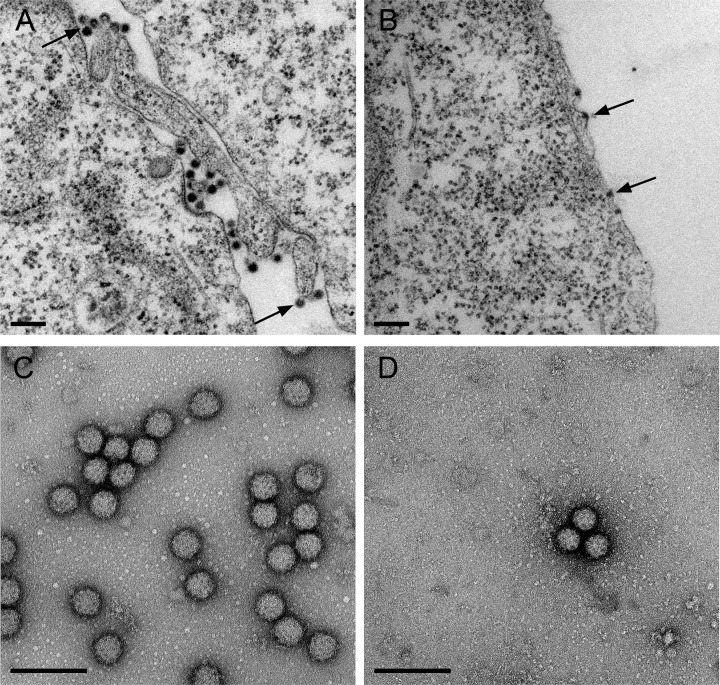
Transmission electron microscopy of WT- and H348/352A-infected cells and virus particles. (A and B) TEM analysis of infected cells. BHK cells were infected with WT SFV or the H348/352A mutant for 7 h and were fixed and processed for TEM. (A) WT-infected cells show nucleocapsids under the plasma membrane (arrows) and abundant budding or released virus particles. (B) H348/352A-infected cells show no released or budding virus particles. Nucleocapsids are visible at the plasma membrane (arrows). (C and D) Negative stain of released virus particles. BHK cells were infected with WT SFV or the H348/352A mutant for 12 h. Virus released in the medium was pelleted through a sucrose cushion and analyzed by negative staining and TEM. Scale bars in panels A to D represent 200 nm.

Transmission electron microscopy (TEM) analysis of WT- and H348/352A-infected cells was used to directly visualize virus particle assembly. The WT virus sample showed nucleocapsids at the plasma membrane in the process of budding and numerous released virus particles (Fig. 6A and Fig. S2A and C). In contrast, H348/352A nucleocapsids associated with the plasma membrane, but little or no apparent evidence of budding or released virus particles was observed (Fig. 6B and Fig. S2B and D). Immunofluorescence analysis showed similar diffuse localization of capsid protein in the cytoplasm and intercellular extensions of WT- and H348/352A-infected cells (Fig. S3). In addition, gradient sedimentation studies showed comparable levels of cytoplasmic nucleocapsid production by the WT and the H348/352A mutant (Fig. S4). These data suggest that the mutant budding defect does not involve E2-capsid interactions or indirect effects on nucleocapsid formation, since nucleocapsids were produced and localized to the plasma membrane.

10.1128/mBio.01564-17.2 FIG S2  Transmission electron microscopy of WT- and H348/352A-infected cells. BHK cells were infected with WT SFV (A and C) or with the H348/352A mutant (B and D) and processed for TEM as described for Fig. 6A and B. Arrows indicate nucleocapsids at the plasma membrane (A to D). Replication complexes (not shown) and replication complex spherules were observed in both WT- and mutant-infected cells. WT-infected cells also show numerous budded virus particles (A and C). Scale bars represent 200 nm. Download FIG S2, JPG file, 2.1 MB. Copyright © 2017 Byrd and Kielian. 2017 Byrd and Kielian This content is distributed under the terms of the Creative Commons Attribution 4.0 International license.

10.1128/mBio.01564-17.3 FIG S3  Confocal microscopy of capsid protein in infected cells. Vero cells were infected with WT SFV or the H348/352A mutant for 8 h; fixed; permeabilized; stained with antibodies to actin, E1/E2, and capsid; and imaged by confocal microscopy. Abundant capsid staining is observed in the cytoplasm and in the intercellular extensions of both WT- and H348/352A-infected cells. Scale bars represent 20 μm. Images are representative of results of 2 independent experiments. Download FIG S3, JPG file, 2.3 MB. Copyright © 2017 Byrd and Kielian. 2017 Byrd and Kielian This content is distributed under the terms of the Creative Commons Attribution 4.0 International license.

10.1128/mBio.01564-17.4 FIG S4  Gradient analysis of cytoplasmic nucleocapsids. BHK cells were infected with WT SFV or the H348/352A mutant for 5 h at 37°C and then labeled for 14 h with [^35^S]methionine/cysteine at 28°C. Cells were lysed in NP-40-containing buffer and analyzed by sucrose gradient sedimentation. Gradient fractions were collected and aliquots were analyzed by SDS-PAGE (A) or scintillation counting (B). Fraction 1 represents the top of the gradient. The capsid protein peak at fraction 11 represents capsid bound to ribosomes, and the peak at fractions 17 to 19 represents cytoplasmic nucleocapsids (26, 49, 61). Results shown are representative of 3 independent experiments. Download FIG S4, JPG file, 0.4 MB. Copyright © 2017 Byrd and Kielian. 2017 Byrd and Kielian This content is distributed under the terms of the Creative Commons Attribution 4.0 International license.

### Morphology and stability of H348/352A virus particles.

**FIG 7:**
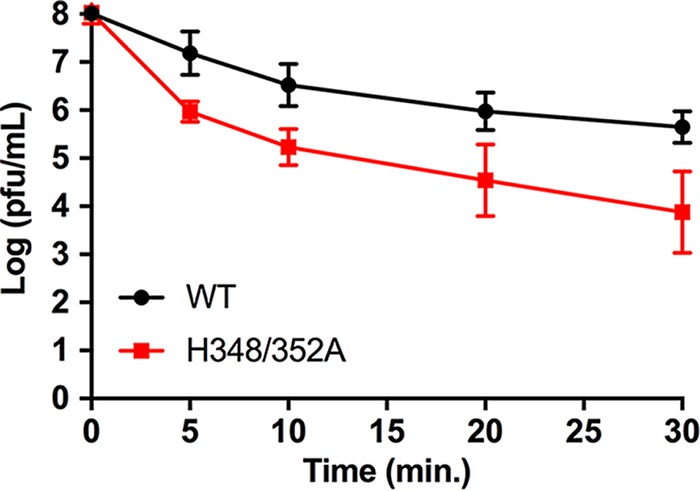
WT and mutant virus thermostability. Stocks of WT and H348/352A mutant virus were incubated at 50°C for the indicated times and the number of infectious particles determined by plaque assay. The graph shows averages and standard deviations of data from 4 independent experiments. The *P* values from 4 independent sample *t* tests for each of the 4 comparisons described above were 0.002, 0.002, 0.0006, and 0.00005 (left to right, respectively). Using a Bonferroni-corrected alpha of 0.0125 for each test, all 4 were statistically significant.

We also used negative-stain electron microscopy to compare WT and mutant virus particles pelleted from the culture medium. This analysis showed that although H348/352A-infected cells produced many fewer virus particles than WT-infected cells, the mutant virus particles had apparently normal morphology (Fig. 6C versus D), in keeping with their unaltered specific infectivity. However, conformational differences in virus glycoproteins can affect the stability of the virus particle, which can have significant implications in virus pathogenesis and virulence (37). To compare the levels of WT and mutant stability, we incubated WT and mutant virus stocks at 50°C for 0 to 30 min and quantitated their infectivity after temperature treatment. While both viruses were increasingly inactivated by elevated temperature over time, the H348/352A mutant was significantly less stable than the WT virus, with a 2-log decrease in infectivity after only 5 min (Fig. 7). Thus, in addition to the mutant budding defect, alanine substitutions of H348 and H352 decrease virus particle stability.

## DISCUSSION

We report here on the role of the conserved histidine residues in the juxtamembrane D-loop of the *Alphavirus* E2 protein. Both single and double alanine substitutions of H348 and H352 caused a significant decrease in virus growth. Detailed analysis of the H348/352A mutant showed that E2/E1 synthesis, dimerization, processing, and transport, as well as virus fusion and specific infectivity were not significantly affected. Although the hypothesis suggested by the virus structure was that H348 and H352 are involved in the low-pH-dependent dissociation of E1 and E2 during virus fusion (23), our data indicated that H348 and H352 do not have an important role in virus entry and fusion. Electron microscopy studies of mutant-infected cells showed that viral nucleocapsids were associated with the plasma membrane, diagnostic of E2-capsid interaction. However, there was little or no evidence for budding or release of virus particles. Thus, the major effect of the mutations was to inhibit a late stage in the virus exit pathway.

Negative-stain electron microscopy studies of released virus particles showed that the WT and mutant viruses had comparable spherical morphologies, with spike complexes visible on the virus envelope. We did note that even after adjusting dilutions to compensate for the reduced production of mutant virus, it was relatively difficult to visualize intact H348/352A particles. Temperature inactivation studies at 50°C showed that the H348/352A mutant was significantly more thermolabile, suggesting that the mutations also affect the architecture and stability of the mature virus particle.

**FIG 8:**
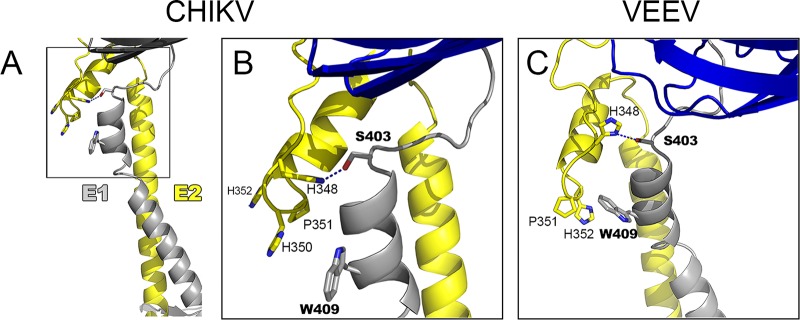
HS348 and H352 interact with E1 residues. Shown are segments corresponding to the stem region of E1 (gray) and E2 (yellow) extracted from the atomic model built for the *Alphavirus* surface glycoprotein shell from CHIKV (A and B) and VEEV (C). A portion of E1 domain III in the glycoprotein layer is shown in blue, as a guide. The stem segments were modeled directly on the cryo-EM reconstructions of CHIKV VLPs to 5-Å resolution (PDB accession no. 3J2W) and of VEEV virions to 4.8-Å resolution (PDB accession no. 3J0C). (A) CHIKV E1 and E2 with the D-loop shown in the boxed region. (B) Expanded view of the CHIKV D-loop from panel A. E2-H348 is in position to form a hydrogen bond with E1-S403. E2-H350 stacks against E1-W409. The positioning of E2-H350 and E2-P351 helps to orientate the juxtamembrane helices N-terminal to the E2 transmembrane domain. Note that SFV residue numbering is used here for CHIKV E2. (C) Expanded view of the D-loop in the VEEV structure. E1 is shown in gray and blue and E2 in yellow. E2-H348 is in position to make a polar interaction with E1-S403. E2-H352 stacks against E1-W409 and together with E2-P351 helps to orientate the juxtamembrane helices N-terminal to the E2 transmembrane domain. Note that SFV residue numbering is used here for VEEV E2. The E2 residue numbering for CHIKV and VEEV is H349, P352, and H353 (see Fig. 1C). The E1 residue numbering is the same for SFV, CHIKV, and VEEV, and E1 residues S403 and W409 are highly conserved. Although the coordinates derived from the 4.8-Å to 5-Å maps are not very accurate, the fact that the same residues were found in interactions in two independent maps, together with the functional data provided here, suggests that these interactions are real. All images were prepared using PyMOL software (the PyMOL Molecular Graphics System, v1.2r2; Schrödinger, LLC).

Because the mutant is blocked at a stage of virus assembly after E2-nucleocapsid interaction at the plasma membrane, we propose that the E2 D-loop is important for correct formation of the E2/E1 lattice during budding. To analyze this possibility further, we considered the previously described atomic models of the envelope protein shell derived from cryo-EM reconstructions of Chikungunya virus-like particles (CHIKV VLPs, see reference 38) and VEEV (see reference 23). The CHIKV VLP model reveals contacts between the E2 D-loop and the E1 protein stem region, with E2-H348 interacting with E1-S403 and E2-H350 stacking against E1-W409 (see details in Fig. 8A and B). The VEEV model shows similar interactions, with E2-H348 interacting with E1-S403 and E2-H352 stacking against E1-W409 (Fig. 8C). While the sequence of the membrane-proximal E1 stem region is not generally conserved, the two interacting residues E1-S403 and E1-W409 are highly conserved (see, e.g., reference 9). In spite of the limits in the resolution of the CHIKV and VEEV maps, the predicted interactions of the E2 D-loop and the conserved E1 stem residues are very similar between the two viruses, supporting the idea of a functional role in establishing the glycoprotein lattice. These models may also explain the apparent lack of additive effects of the E2-H348A and E2-H352A mutations in SFV, as there may be some plasticity in the interactions of the D-loop, with the SFV E2-W350 being able to stack with E1-W409, similarly to the stacking of E2-H350 with E1-W409 in CHIKV.

Previous studies of a Sindbis virus mutant, *ts*103, showed that it is inhibited at a late stage of virus budding and produces aberrant multicored particles that are less thermostable (39). The *ts*103 phenotype is due to a mutation of E2-A344 to V (40), a position at the N-terminal boundary of E2 subdomain D (23). Unlike H348 and H352, A344 is not highly conserved (Fig. 1C) and does not appear to make specific contacts with E1. While the effects of the E2-A344V mutation may be more indirect than those of the D-loop mutations, its partial rescue by a second-site mutation in the membrane-distal tip of E1 (40) is also consistent with effects on the E2/E1 lattice.

The envelope and capsid proteins both form organized lattices during *Alphavirus* assembly. These lattices are connected via the binding of a Tyr-X-Leu motif in the E2 cytoplasmic domain to the capsid hydrophobic pocket (14, 15), an interaction that is required for virus budding (13, 41). The functional importance of the capsid lattice versus the E2/E1 lattice during assembly is not clear. The capsid protein can assemble with RNA into nucleocapsid-like structures *in vitro* (42–45). Nucleocapsids can also assemble in the cytoplasm in the absence of envelope protein expression or virus infection (46, 47). However, while self-assembly information is clearly contained within the capsid protein, under infection conditions there is evidence that nucleocapsid formation is regulated by the expression of the envelope proteins and the E2 cytoplasmic domain (47). Moreover, capsid mutants that block capsid-capsid interactions and cytoplasmic nucleocapsid assembly are still able to bud at the plasma membrane, where the interaction with the E2/E1 lattice through the E2 cytoplasmic domain is sufficient to drive formation of the nucleocapsid (48, 49). There is also evidence that cytoplasmic nucleocapsids undergo additional structural changes or maturation in response to glycoprotein binding during budding (26, 50). To add to the complexity of *Alphavirus* assembly, expression of the small hydrophobic proteins 6K and transframe appears to promote budding at the plasma membrane (24, 51), although their relative roles and mechanisms are unclear. While many viruses use host cell machinery such as the endosomal sorting complexes required for transport (ESCRT) pathway to promote budding (52), *Alphavirus* budding is ESCRT independent (53). The role of other host proteins, however, is largely unknown.

While it is clear that many unanswered questions on *Alphavirus* assembly and budding remain, our work identifies new interactions in E2 and E1 that promote virus budding. The E2 D-loop residues H348 and H352 have specific interactions with the E1 stem, particularly with respect to residues S403 and W409. While the D-loop did not show the hypothesized function in dimer dissociation during entry, its interaction with its E1 dimeric partner proved important in promoting virus exit. Our results suggest that the E2 D-loop has a significant role in formation of the glycoprotein lattice necessary for membrane curvature and *Alphavirus* budding, highlighting the importance of E1-E2 lateral protein interactions in *Alphavirus* exit.

## MATERIALS AND METHODS

### Cells and viruses.

BHK-21 cells were propagated in complete BHK medium (Dulbecco’s modified Eagle’s medium [DMEM] plus 5% fetal bovine serum, 10% tryptose phosphate broth, and 100 U penicillin/ml and 100 μg streptomycin/ml) at 37°C unless otherwise noted. All plaque assays were performed using BHK cells. Vero cells were cultured at 37°C in DMEM plus 10% fetal bovine serum and 100 U penicillin/ml and 100 μg streptomycin/ml.

Mutant viruses were created by site-directed mutagenesis of the DG-1 plasmid, a subclone of the pSP6-SFV4 infectious clone (24), as previously described (54). In summary, mutations were introduced into the DG-1 plasmid using PrimeSTAR HS DNA polymerase (TaKaRa Bio Company, Kusatsu, Japan) using the following primers: for H348A, 5′ CCACTGAAGGGAAACCGGCCGGCTGGC 3′ (forward) and 5′ CTGATGCGGCCAGCCGGCCGGTTTCCC 3′ (reverse); for H352A, 5′ CCGCACGGCTGGCCGGCTCAGATCGTAC 3′ (forward) and 5′ GTACTGTACGATCTGAGCCGGCCAGCCGTG 3′ (reverse); and for H348/352A, 5′ GGGAAACCGGCCGGCTGGCCGGCTCAG 3′ (forward) and 5′ GTACGATCTGAGCCGGCCAGCCGGCCGG 3′ (reverse). DG-1 clones containing the correct mutation as validated by sequencing were subcloned into the pSP6-SFV4 infectious clone using the NsiI and SpeI restriction endonuclease sites. Infectious clones were verified by sequencing of the entire NsiI/SpeI fragment (Genewiz, South Plainfield, NJ). Infectious clone RNA was generated by *in vitro* transcription (24). Virus stocks were prepared by electroporation of BHK cells with infectious clone RNA and collection of the cell media at 24 h postelectroporation. For each mutant, 2 infectious clones were independently generated and sequenced and used to verify results as indicated.

### Immunofluorescence.

BHK cells were electroporated with WT or mutant RNA, seeded on coverslips, and incubated at 37°C for 8 h. Cells were washed with phosphate-buffered saline (PBS) and fixed with 4% paraformaldehyde (PFA) at room temperature for 20 min. Cells were permeabilized in 0.02% Triton X-100 when appropriate and stained with primary antibodies, including a pAb against E1 and E2, or with MAbs specific for E1 or E2 (55). Cells were then stained with appropriate secondary antibodies conjugated with Alexa Fluor 488 (Molecular Probes) and with Hoechst 33342 nuclear stain (Invitrogen). Images were acquired by immunofluorescence microscopy.

### Virus growth assays.

BHK cells were electroporated with WT or mutant RNA, mixed with an equal concentration of nonelectroporated cells, plated in 6-well culture dishes, and incubated at 37°C. After 2 h of incubation, cells were washed once with infection media (minimal essential medium [MEM] with 0.2% bovine serum albumin [BSA], 100 U penicillin/ml, 100 μg streptomycin/ml, and 10 mM HEPES [pH 8.0]) and incubated at 37°C or 28°C in fresh media. At the indicated time points, the cell culture media were collected and pelleted at 20.8 K × *g* for 10 min to remove cell debris, and the titer was determined by plaque assay. Where indicated, statistics were calculated by 2-way analysis of variance (ANOVA) with Tukey’s multiple-comparison test using Prism (56).

### pH dependence of fusion.

BHK cells were prebound with a virus multiplicity of infection (MOI) of ~0.2 PFU/cell for 90 min on ice to prevent endocytosis. The cells were then treated for 3 min at 37°C with media buffered at the indicated pH to trigger virus fusion with the plasma membrane. Cells were incubated at 28°C for 16 to 18 h in complete BHK media containing 20 mM NH_4_Cl to prevent secondary infection and were scored for infection by immunofluorescence to determine the percentages of infected cells, which were comparable between the WT and the mutants. Statistical significance was determined by 2-way ANOVA with Tukey’s multiple-comparison test using Prism (56).

### Specific infectivity.

BHK cells were infected with WT or H348/352A virus at an MOI of 10, incubated at 37°C, and washed at 1 h postinfection with infection medium. At 8 hpi, supernatants were collected and centrifuged to remove cell debris, and an aliquot was saved for plaque assay. The remaining supernatant was layered over a 20% sucrose cushion and centrifuged at 35 K rpm for 3 h at 4°C using an SW 41 rotor. Pellets were resuspended identically in TN buffer (100 mM NaCl and 50 mM Tris, pH 7.4) containing 10 μg/ml leupeptin, 100 μg/ml pepstatin, 0.5 mM phenylmethylsulfonyl fluoride (PMSF), and 20 μg/ml aprotinin and incubated on ice overnight. WT virus samples were diluted 1:100 to account for ~2-log reduction in growth of H348/352A. Virus suspensions were serially diluted in TN buffer with protease inhibitors, heated to 95°C with SDS sample buffer, and analyzed by SDS-PAGE. Gels were transferred to nitrocellulose membranes and blotted with MAb to E2 and Alexa Fluor 680-conjugated secondary Ab. The amount of E2 in each dilution was then quantitated using a Li-COR Odyssey Classic fluorescence imaging scanner (Li-COR Biosciences). The specific infectivity was calculated as the ratio of the virus infectivity to the concentration of E2 (number of virus particles). Statistical significance was calculated by unpaired *t* test using Prism software (56).

### Extension induction and capsid expression.

Vero cells plated on MatTek glass-bottom chambers were infected with 5 PFU/cell of WT or H348/352A virus. Cells were incubated at 37°C and fixed with 4% PFA at 8 hpi, permeabilized with 0.02% Triton X-100, and stained with the appropriate Abs (anti-tubulin, E1 and E2 or 568 Phalloidin, E1/E2 pAb, and capsid Ab). Cells were imaged by confocal microscopy using an LSM5 Live DuoScan confocal microscope (Carl Zeiss MicroImaging, Inc.).

### Assembly and dimer stability assays.

BHK cells were infected with 10 PFU/cell of WT or H348/352A virus and incubated at 37°C. At 4.5 hpi, cell media were changed to MEM without methionine and cysteine and cells were starved for 20 min. At 5 hpi, cells were pulse-labeled with 100 μCi/ml [^35^S]methionine/cysteine for 30 min at 37°C. Following the pulse-labeling, cells were washed with media containing a 10× excess of cysteine and methionine and were chased for the indicated times. For assembly assays, supernatants were collected and immunoprecipitated with an E1/E2 pAb in the absence of detergent to allow retrieval of intact virus particles. Cells were lysed in TN buffer containing 1% Triton X-100, 1 mM EDTA, 1 μg/ml pepstatin, 50 μg/ml leupeptin, 1 mg/ml BSA, 1% aprotinin, and 1 mM PMSF, and the lysates were immunoprecipitated with an E1/E2 pAb and washed with radioimmunoprecipitation assay (RIPA) buffer (57) or were coimmunoprecipitated with an E1 MAb (dimer dissociation assay) using a modified RIPA buffer without SDS and Na-deoxycholate. Samples were evaluated by SDS-PAGE and imaged using a Storm 860 Phosphorimager (Molecular Dynamics).

### Electron microscopy.

BHK cells were infected with virus at an MOI of 10 and incubated at 37°C. At 7 hpi, cells were fixed for 30 min at room temperature with 2.5% glutaraldehyde–2% paraformaldehyde–0.1 M cacodylate buffer and were embedded and processed by the Einstein Analytical Imaging Facility for transmission electron microscopy. Images were acquired on a JEOL 1200X electron microscope.

To image virus by negative staining, BHK cells were infected at an MOI of 10 and incubated at 37°C for 12 h. Cell media were harvested, centrifuged to remove cellular debris, and pelleted through a 20% sucrose cushion as described above. Virus pellets were resuspended in 20 mM Tris (pH 7.4), adsorbed to glow-discharged electron microscopy grids, and stained with uranyl acetate. Samples were imaged on a JEOL 1400Plus TEM (SIG no. 1S10OD016214).

### Virus thermostability.

Virus stocks were diluted in infection media to 1 × 10^8^ PFU/ml, incubated for the indicated times at 50°C, and immediately placed on ice. Infectious virus was quantitated by plaque assay. Statistical significance was determined by performing multiple *t* tests using Prism (56) and a Bonferroni-corrected alpha.

### Nucleocapsid gradients.

Cytoplasmic nucleocapsids were analyzed by sucrose gradient sedimentation as previously described (26, 49) with minor adjustments. BHK cells were infected with 10 PFU/cell and incubated at 37°C for 5 h. Cells were labeled with 50 μCi/ml [^35^S]methionine/cysteine for 14 h at 28°C, followed by a brief (30-min) chase. Cells were lysed (100 mM Tris [pH 7.4], 50 mM NaCl, 2 mM EDTA, 1% NP-40, 40 μM N-ethylmaleimide, 250 U/ml RNase inhibitor, 1 μg/ml pepstatin, 5 μg/ml leupeptin, 1 mM PMSF, and 1% aprotinin) and pelleted at 20.8 K × *g* for 10 min to remove cell nuclei. Lysates were then incubated on ice for 20 min with 25 mM EDTA to dissociate polysomes. Samples were loaded onto 7.5% to 20% (wt/wt) linear sucrose gradients in TN buffer with 2 mM EDTA and 0.1% NP-40 and centrifuged at 41 K rpm for 2 h at 4°C in an SW41 rotor. Fractions of approximately 500 μl were collected and analyzed by SDS-PAGE and scintillation counting. Gels were imaged using a Storm 860 Phosphorimager (Molecular Dynamics).

## References

[1] Kuhn RJ 2013 Togaviridae, p 629-650. *In* Knipe DM, Howley PM, Cohen JI, Griffin DE, Lamb RA, Martin MA, Racaniello VR, Roizman B (ed), Fields virology, 6th ed, vol. 1 Lippincott Williams & Wilkins, Philadelphia, PA.

[2] Griffin DE 2013 Alphaviruses, p 651-686. *In* Knipe DM, Howley PM, Cohen JI, Griffin DE, Lamb RA, Martin MA, Racaniello VR, Roizman B (ed), Fields virology, 6th ed, vol. 1 Lippincott Williams & Wilkins, Philadelphia, PA.

[3] Weaver SC, Winegar R, Manger ID, Forrester NL 2012 Alphaviruses: population genetics and determinants of emergence. Antiviral Res 94:242–257. doi:10.1016/j.antiviral.2012.04.002. 22522323 PMC3737490

[4] Schwartz O, Albert ML 2010 Biology and pathogenesis of Chikungunya virus. Nat Rev Microbiol 8:491–500. doi:10.1038/nrmicro2368. 20551973

[5] Enserink M 2014 Crippling virus set to conquer Western hemisphere. Science 344:678–679. doi:10.1126/science.344.6185.678. 24833366

[6] Johansson MA 2015 Chikungunya on the move. Trends Parasitol 31:43–45. doi:10.1016/j.pt.2014.12.008. 25649340 PMC4583061

[7] Morrison TE 2014 Reemergence of Chikungunya virus. J Virol 88:11644–11647. doi:10.1128/JVI.01432-14. 25078691 PMC4178719

[8] Jose J, Snyder JE, Kuhn RJ 2009 A structural and functional perspective of Alphavirus replication and assembly. Future Microbiol 4:837–856. doi:10.2217/fmb.09.59. 19722838 PMC2762864

[9] Voss JE, Vaney MC, Duquerroy S, Vonrhein C, Girard-Blanc C, Crublet E, Thompson A, Bricogne G, Rey FA 2010 Glycoprotein organization of Chikungunya virus particles revealed by X-ray crystallography. Nature 468:709–712. doi:10.1038/nature09555. 21124458

[10] Kielian M 2010 Structural biology: an Alphavirus puzzle solved. Nature 468:645–646. doi:10.1038/468645a. 21124448 PMC3088109

[11] Kielian M 2014 Mechanisms of virus membrane fusion proteins. Annu Rev Virol 1:171–189. doi:10.1146/annurev-virology-031413-085521. 26958720

[12] Kielian M, Rey FA 2006 Virus membrane fusion proteins: more than one way to make a hairpin. Nat Rev Microbiol 4:67–76. doi:10.1038/nrmicro1326. 16357862 PMC7097298

[13] Zhao H, Lindqvist B, Garoff H, von Bonsdorff CH, Liljeström P 1994 A tyrosine-based motif in the cytoplasmic domain of the Alphavirus envelope protein is essential for budding. EMBO J 13:4204–4211. 7925266 10.1002/j.1460-2075.1994.tb06740.x PMC395347

[14] Skoging U, Vihinen M, Nilsson L, Liljeström P 1996 Aromatic interactions define the binding of the Alphavirus spike to its nucleocapsid. Structure 4:519–529. doi:10.1016/S0969-2126(96)00058-5. 8736551

[15] Lee S, Owen KE, Choi HK, Lee H, Lu G, Wengler G, Brown DT, Rossmann MG, Kuhn RJ 1996 Identification of a protein binding site on the surface of the Alphavirus nucleocapsid and its implication in virus assembly. Structure 4:531–541. doi:10.1016/S0969-2126(96)00059-7. 8736552

[16] Li L, Jose J, Xiang Y, Kuhn RJ, Rossmann MG 2010 Structural changes of envelope proteins during Alphavirus fusion. Nature 468:705–708. doi:10.1038/nature09546. 21124457 PMC3057476

[17] Sánchez-San Martín C, Liu CY, Kielian M 2009 Dealing with low pH: entry and exit of alphaviruses and flaviviruses. Trends Microbiol 17:514–521. doi:10.1016/j.tim.2009.08.002. 19796949 PMC2783195

[18] Wahlberg JM, Boere WAM, Garoff H 1989 The heterodimeric association between the membrane proteins of Semliki Forest virus changes its sensitivity to low pH during virus maturation. J Virol 63:4991–4997. 2479769 10.1128/jvi.63.12.4991-4997.1989 PMC251158

[19] Sjöberg M, Lindqvist B, Garoff H 2011 Activation of the Alphavirus spike protein is suppressed by bound E3. J Virol 85:5644–5650. doi:10.1128/JVI.00130-11. 21430054 PMC3094962

[20] Uchime O, Fields W, Kielian M 2013 The role of E3 in pH protection during Alphavirus assembly and exit. J Virol 87:10255–10262. doi:10.1128/JVI.01507-13. 23864626 PMC3754015

[21] Salminen A, Wahlberg JM, Lobigs M, Liljeström P, Garoff H 1992 Membrane fusion process of Semliki Forest virus II: cleavage-dependent reorganization of the spike protein complex controls virus entry. J Cell Biol 116:349–357. doi:10.1083/jcb.116.2.349. 1730759 PMC2289290

[22] Fields W, Kielian M 2013 A key interaction between the Alphavirus envelope proteins responsible for initial dimer dissociation during fusion. J Virol 87:3774–3781. doi:10.1128/JVI.03310-12. 23325694 PMC3624238

[23] Zhang R, Hryc CF, Cong Y, Liu X, Jakana J, Gorchakov R, Baker ML, Weaver SC, Chiu W 2011 4.4 A cryo-EM structure of an enveloped Alphavirus Venezuelan equine encephalitis virus. EMBO J 30:3854–3863. doi:10.1038/emboj.2011.261. 21829169 PMC3173789

[24] Liljeström P, Lusa S, Huylebroeck D, Garoff H 1991 In vitro mutagenesis of a full-length cDNA clone of Semliki Forest virus: the small 6,000-molecular-weight membrane protein modulates virus release. J Virol 65:4107–4113. 2072446 10.1128/jvi.65.8.4107-4113.1991 PMC248843

[25] Duffus WA, Levy-Mintz P, Klimjack MR, Kielian M 1995 Mutations in the putative fusion peptide of Semliki Forest virus affect spike protein oligomerization and virus assembly. J Virol 69:2471–2479. 7884895 10.1128/jvi.69.4.2471-2479.1995 PMC188922

[26] Zheng Y, Kielian M 2015 An Alphavirus temperature-sensitive capsid mutant reveals stages of nucleocapsid assembly. Virology 484:412–420. doi:10.1016/j.virol.2015.05.011. 26051211 PMC4567448

[27] Wahlberg JM, Garoff H 1992 Membrane fusion process of Semliki Forest virus I: low pH-induced rearrangement in spike protein quaternary structure precedes virus penetration into cells. J Cell Biol 116:339–348. doi:10.1083/jcb.116.2.339. 1370493 PMC2289294

[28] Glomb-Reinmund S, Kielian M 1998 fus-1, a pH-shift mutant of Semliki Forest virus, acts by altering spike subunit interactions via a mutation in the E2 subunit. J Virol 72:4281–4287. 9557718 10.1128/jvi.72.5.4281-4287.1998 PMC109658

[29] Zhang X, Kielian M 2005 An interaction site of the envelope proteins of Semliki Forest virus that is preserved after proteolytic activation. Virology 337:344–352. doi:10.1016/j.virol.2005.04.021. 15913697

[30] Liu CY, Kielian M 2009 E1 mutants identify a critical region in the trimer interface of the Semliki Forest virus fusion protein. J Virol 83:11298–11306. doi:10.1128/JVI.01147-09. 19692469 PMC2772772

[31] Martinez MG, Snapp EL, Perumal GS, Macaluso FP, Kielian M 2014 Imaging the Alphavirus exit pathway. J Virol 88:6922–6933. doi:10.1128/JVI.00592-14. 24696489 PMC4054368

[32] Martinez MG, Kielian M 2016 Intercellular extensions are induced by the Alphavirus structural proteins and mediate virus transmission. PLoS Pathog 12:e1006061. doi:10.1371/journal.ppat.1006061. 27977778 PMC5158078

[33] Jose J, Tang J, Taylor AB, Baker TS, Kuhn RJ 2015 Fluorescent protein-tagged Sindbis virus E2 glycoprotein allows single particle analysis of virus budding from live cells. Viruses 7:6182–6199. doi:10.3390/v7122926. 26633461 PMC4690852

[34] Jose J, Taylor AB, Kuhn RJ 2017 Spatial and temporal analysis of Alphavirus replication and assembly in mammalian and mosquito cells. mBio 8:e02294-16. doi:10.1128/mBio.02294-16. 28196962 PMC5312085

[35] Zhao H, Garoff H 1992 Role of cell surface spikes in Alphavirus budding. J Virol 66:7089–7095. 1331511 10.1128/jvi.66.12.7089-7095.1992 PMC240383

[36] Lu YE, Eng CH, Shome SG, Kielian M 2001 In vivo generation and characterization of a soluble form of the Semliki Forest virus fusion protein. J Virol 75:8329–8339. doi:10.1128/JVI.75.17.8329-8339.2001. 11483778 PMC115077

[37] Kostyuchenko VA, Lim EX, Zhang S, Fibriansah G, Ng TS, Ooi JS, Shi J, Lok SM 2016 Structure of the thermally stable Zika virus. Nature 533:425–428. doi:10.1038/nature17994. 27093288

[38] Sun S, Xiang Y, Akahata W, Holdaway H, Pal P, Zhang X, Diamond MS, Nabel GJ, Rossmann MG 2013 Structural analyses at pseudo atomic resolution of Chikungunya virus and antibodies show mechanisms of neutralization. Elife 2:e00435. doi:10.7554/eLife.00435. 23577234 PMC3614025

[39] Strauss EG, Birdwell CR, Lenches EM, Staples SE, Strauss JH 1977 Mutants of Sindbis virus. II. Characterization of a maturation-defective mutant, ts103. Virology 82:122–149. doi:10.1016/0042-6822(77)90038-1. 898673

[40] Hahn CS, Rice CM, Strauss EG, Lenches EM, Strauss JH 1989 Sindbis virus ts103 has a mutation in glycoprotein E2 that leads to defective assembly of virions. J Virol 63:3459–3465. 2746736 10.1128/jvi.63.8.3459-3465.1989 PMC250922

[41] Owen KE, Kuhn RJ 1997 Alphavirus budding is dependent on the interaction between the nucleocapsid and hydrophobic amino acids on the cytoplasmic domain of the E2 envelope glycoprotein. Virology 230:187–196. doi:10.1006/viro.1997.8480. 9143274

[42] Tellinghuisen TL, Hamburger AE, Fisher BR, Ostendorp R, Kuhn RJ 1999 In vitro assembly of Alphavirus cores by using nucleocapsid protein expressed in *Escherichia coli*. J Virol 73:5309–5319. 10364277 10.1128/jvi.73.7.5309-5319.1999 PMC112586

[43] Tellinghuisen TL, Kuhn RJ 2000 Nucleic acid-dependent cross-linking of the nucleocapsid protein of Sindbis virus. J Virol 74:4302–4309. doi:10.1128/JVI.74.9.4302-4309.2000. 10756045 PMC111947

[44] Mukhopadhyay S, Chipman PR, Hong EM, Kuhn RJ, Rossmann MG 2002 In vitro-assembled Alphavirus core-like particles maintain a structure similar to that of nucleocapsid cores in mature virus. J Virol 76:11128–11132. doi:10.1128/JVI.76.21.11128-11132.2002. 12368355 PMC136650

[45] Cheng F, Mukhopadhyay S 2011 Generating enveloped virus-like particles with in vitro assembled cores. Virology 413:153–160. doi:10.1016/j.virol.2011.02.001. 21334709

[46] Akahata W, Yang ZY, Andersen H, Sun S, Holdaway HA, Kong WP, Lewis MG, Higgs S, Rossmann MG, Rao S, Nabel GJ 2010 A virus-like particle vaccine for epidemic Chikungunya virus protects nonhuman primates against infection. Nat Med 16:334–338. doi:10.1038/nm.2105. 20111039 PMC2834826

[47] Snyder JE, Berrios CJ, Edwards TJ, Jose J, Perera R, Kuhn RJ 2012 Probing the early temporal and spatial interaction of the Sindbis virus capsid and E2 proteins with reverse genetics. J Virol 86:12372–12383. doi:10.1128/JVI.01220-12. 22951842 PMC3486501

[48] Forsell K, Xing L, Kozlovska T, Cheng RH, Garoff H 2000 Membrane proteins organize a symmetrical virus. EMBO J 19:5081–5091. doi:10.1093/emboj/19.19.5081. 11013211 PMC302099

[49] Skoging-Nyberg U, Liljeström P 2001 M-X-I motif of Semliki Forest virus capsid protein affects nucleocapsid assembly. J Virol 75:4625–4632. doi:10.1128/JVI.75.10.4625-4632.2001. 11312332 PMC114215

[50] Jose J, Przybyla L, Edwards TJ, Perera R, Burgner JW II, Kuhn RJ 2012 Interactions of the cytoplasmic domain of Sindbis virus e2 with nucleocapsid cores promote Alphavirus budding. J Virol 86:2585–2599. doi:10.1128/JVI.05860-11. 22190727 PMC3302261

[51] Snyder JE, Kulcsar KA, Schultz KL, Riley CP, Neary JT, Marr S, Jose J, Griffin DE, Kuhn RJ 2013 Functional characterization of the Alphavirus TF protein. J Virol 87:8511–8523. doi:10.1128/JVI.00449-13. 23720714 PMC3719798

[52] Votteler J, Sundquist WI 2013 Virus budding and the ESCRT pathway. Cell Host Microbe 14:232–241. doi:10.1016/j.chom.2013.08.012. 24034610 PMC3819203

[53] Taylor GM, Hanson PI, Kielian M 2007 Ubiquitin depletion and dominant-negative VPS4 inhibit rhabdovirus budding without affecting Alphavirus budding. J Virol 81:13631–13639. doi:10.1128/JVI.01688-07. 17913808 PMC2168838

[54] Chanel-Vos C, Kielian M 2004 A conserved histidine in the ij loop of the Semliki Forest virus E1 protein plays an important role in membrane fusion. J Virol 78:13543–13552. doi:10.1128/JVI.78.24.13543-13552.2004. 15564465 PMC533937

[55] Kielian M, Jungerwirth S, Sayad KU, DeCandido S 1990 Biosynthesis, maturation, and acid-activation of the Semliki Forest virus fusion protein. J Virol 64:4614–4624. 2118964 10.1128/jvi.64.10.4614-4624.1990 PMC247945

[56] Ivashchenko R, Bykov I, Datsko A, Dolgaya L, Goodz A, Shayna A 2017 Prism 7 for Mac OS X v7.0c. GraphPad Software, Inc, La Jolla, CA.

[57] Liao M, Kielian M 2006 Functions of the stem region of the Semliki Forest virus fusion protein during virus fusion and assembly. J Virol 80:11362–11369. doi:10.1128/JVI.01679-06. 16971447 PMC1642169

[58] Combet C, Blanchet C, Geourjon C, Deléage G 2000 NPS@: network protein sequence analysis. Trends Biochem Sci 25:147–150. doi:10.1016/S0968-0004(99)01540-6. 10694887

[59] Thompson JD, Higgins DG, Gibson TJ 1994 CLUSTAL W: improving the sensitivity of progressive multiple sequence alignment through sequence weighting, position-specific gap penalties and weight matrix choice. Nucleic Acids Res 22:4673–4680. doi:10.1093/nar/22.22.4673. 7984417 PMC308517

[60] Robert X, Gouet P 2014 Deciphering key features in protein structures with the new ENDscript server. Nucleic Acids Res 42:W320–W324. doi:10.1093/nar/gku316. 24753421 PMC4086106

[61] Ulmanen I, Söderlund H, Kääriäinen L 1976 Semliki Forest virus capsid protein associates with the 60S ribosomal subunit in infected cells. J Virol 20:203–210. 824460 10.1128/jvi.20.1.203-210.1976 PMC354981

