# Supplementary figures and images for: An Alphavirus E2 Membrane-Proximal Domain Promotes Envelope Protein Lateral Interactions and Virus Budding
Source: mBio. 2017 Nov 7;8(6):e01564-17. doi: 10.1128/mBio.01564-17 (PMC5676042; doi:10.1128/mBio.01564-17)

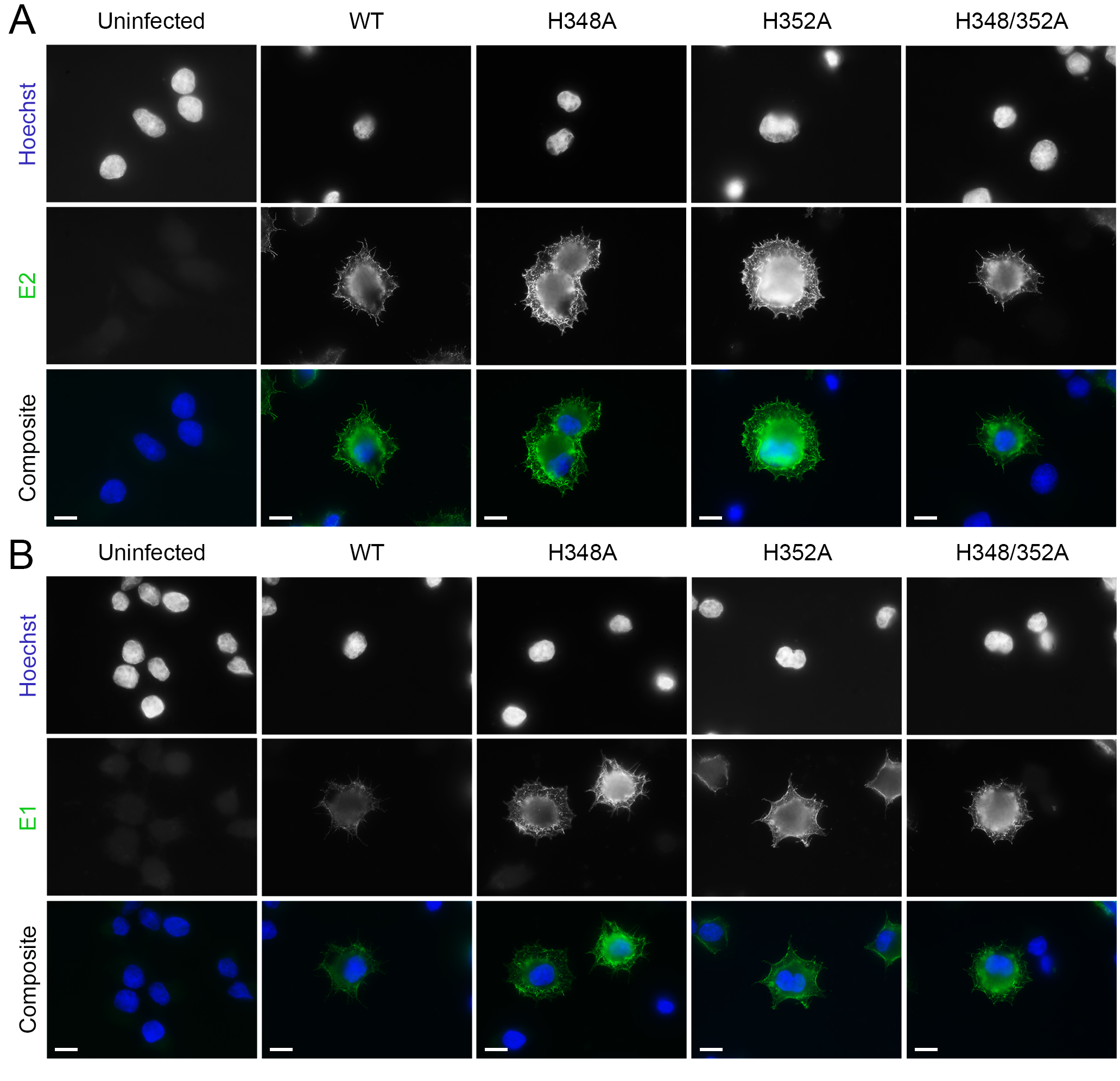

Supplement: FIG S1 [file mbo006173580sf1.jpg]

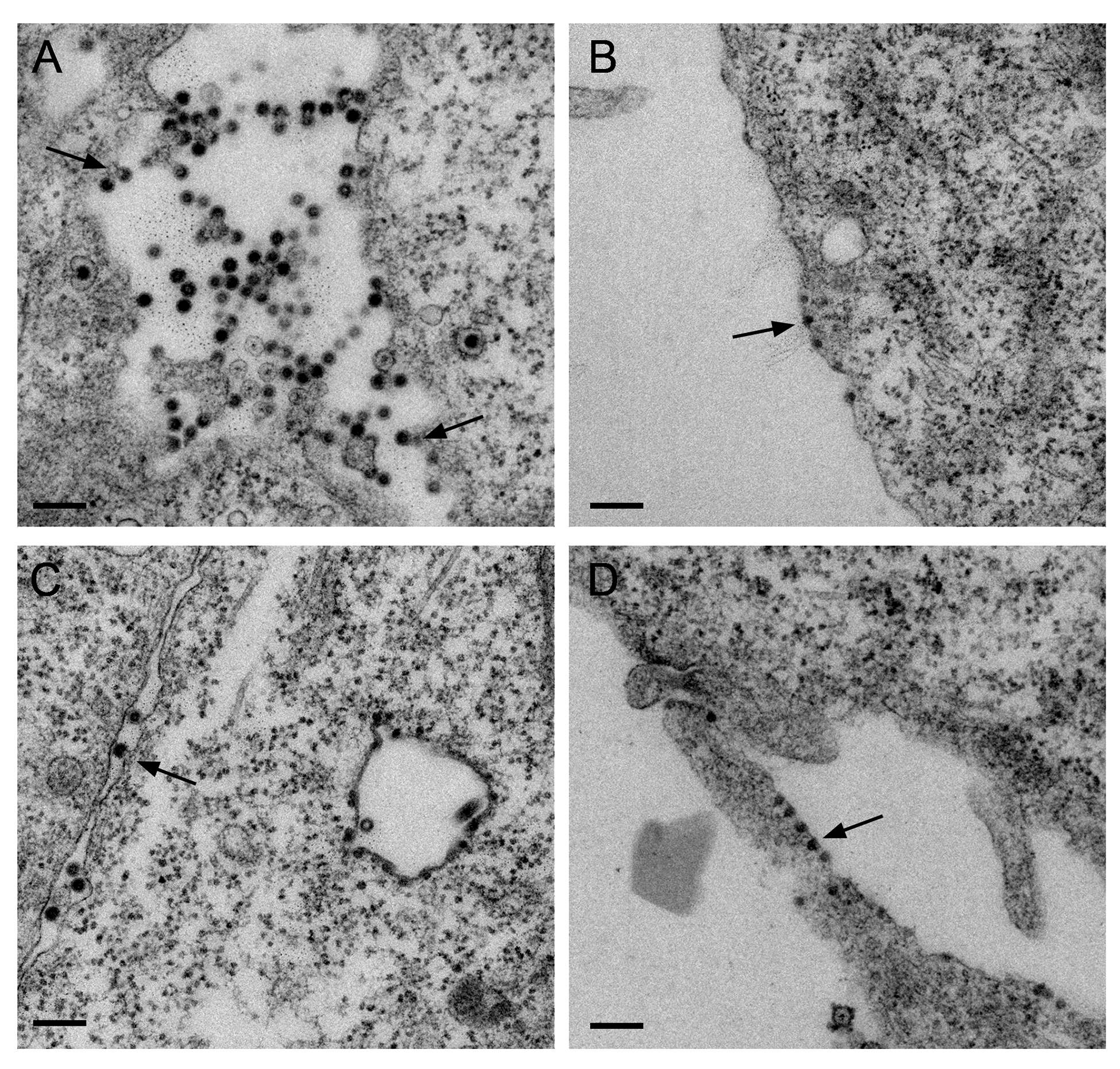

Supplement: FIG S2 [file mbo006173580sf2.jpg]

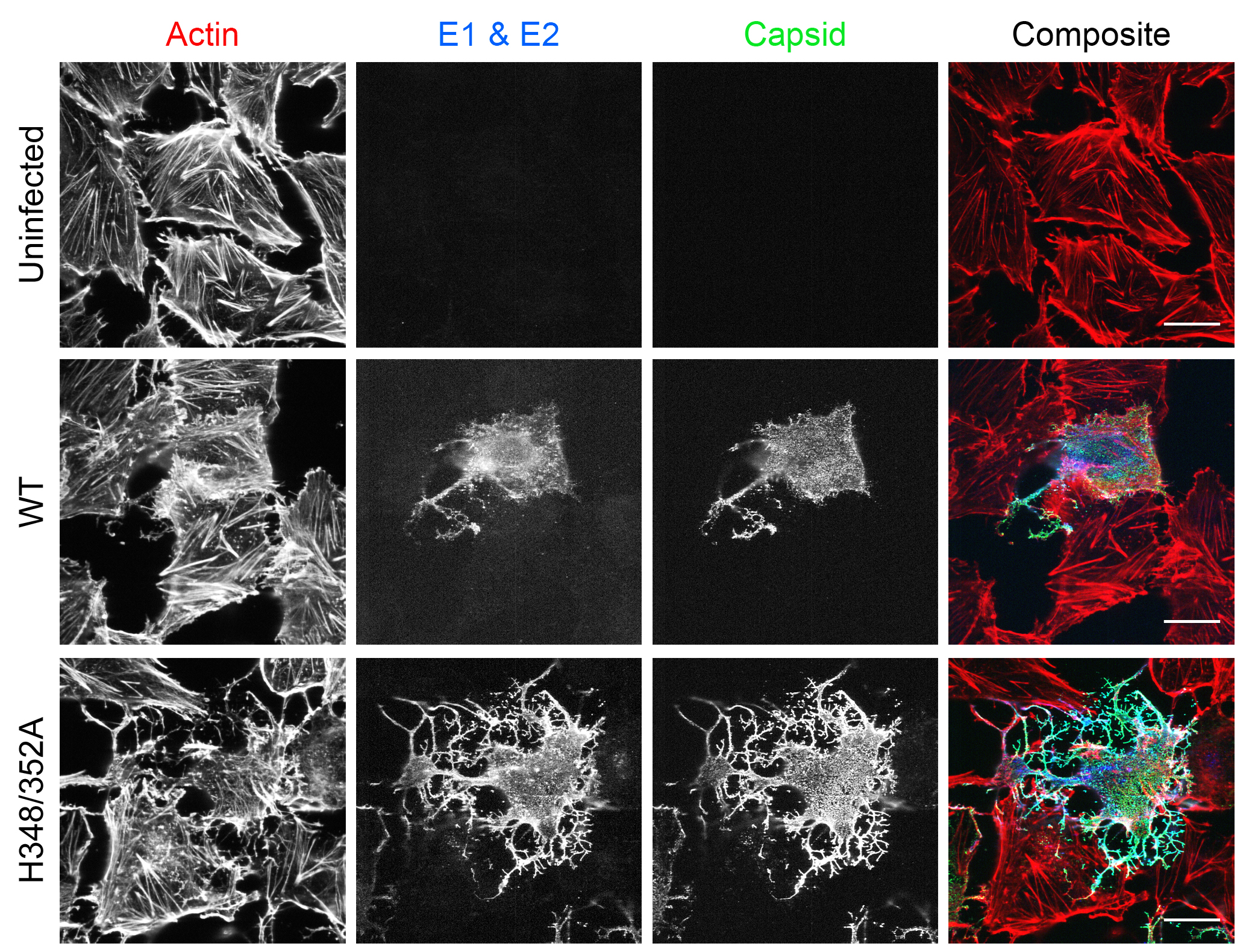

Supplement: FIG S3 [file mbo006173580sf3.jpg]

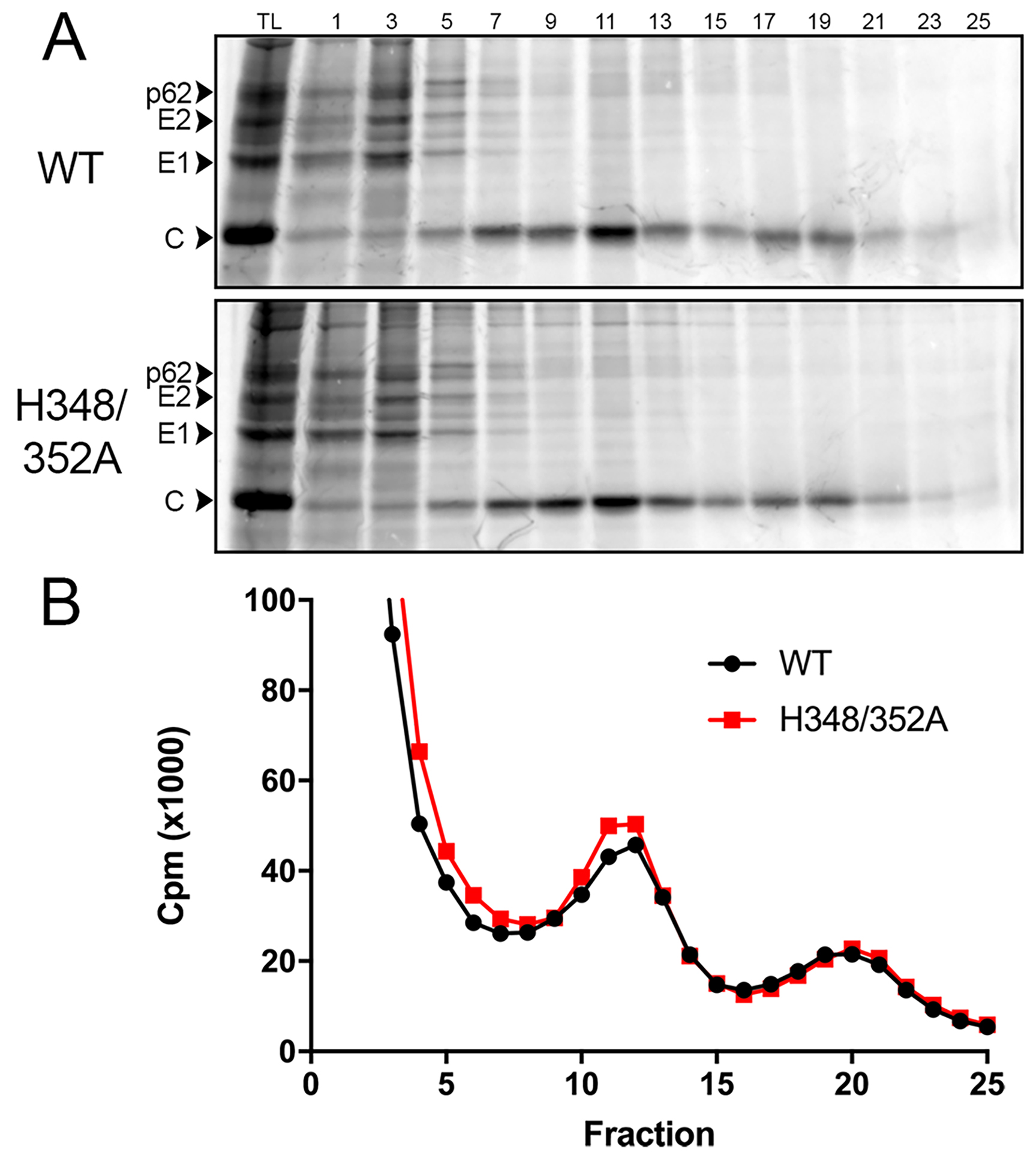

Supplement: FIG S4 [file mbo006173580sf4.jpg]
